# Serum HE4, CA125, YKL-40, bcl-2, cathepsin-L and prediction optimal debulking surgery, response to chemotherapy in ovarian cancer

**DOI:** 10.1186/1757-2215-7-62

**Published:** 2014-06-10

**Authors:** Anita Monika Chudecka-Głaz, Aneta Alicja Cymbaluk-Płoska, Janusz Leszek Menkiszak, Agnieszka Monika Sompolska-Rzechuła, Aleksandra Izabela Tołoczko-Grabarek, Izabella Anna Rzepka-Górska

**Affiliations:** 1 Department of Gynecological Surgery and Gynecological Oncology of Adults and Adolescents, Pomeranian Medical University, Szczecin, Poland; 2 Department of Mathematics Applications in Economy, West Pomeranian University of Technology, Szczecin, Poland; 3 International Hereditary Cancer Centre, Department of Genetics and Pathology, Pomeranian Medical University, Szczecin, Poland

**Keywords:** HE4, CA125, YKL-40, Cathepsin-L, Neoadjuvant chemotherapy, Debulking surgery, Ovarian cancer

## Abstract

**Background:**

The most important prognostic factor in the ovarian cancer is optimal cytoreduction. The neoadjuvant chemotherapy, an only optional method of treatment in this case and is still the subject of debate. The object of this study was to evaluate the usefulness of markers: CA 125, HE4, YKL-40 and bcl-2 as well as cathepsin L in predicting optimal cytoreduction and response to chemotherapy.

**Methods:**

Sera were secured preoperatively. The division into groups was performed retrospectively depending on the method of treatment (surgery vs neoadjuvant chemotherapy) as well as on response to chemotherapy (sensitive vs resistant vs refractory). Comparisons were made between groups, and the diagnostic usefulness of tested proteins was examined.

**Results:**

We found that statistically significant differences between primary operated patients and patients undergoing neoadjuvant chemotherapy were applicable only to the tumour markers (CA125 1206.79 vs 2432.38, p = 0.000191; HE4 78.87 vs 602.45, p = 0.000004; YKL-40 108.13 vs 203.96, p = 0.003991). Cathepsin-L and Bcl-2 were statistically insignificant. The cut-off point values were determined for the CA 125 (345 mIU/ml), HE4 (218.43 pmol/L) and YKL-40 (140.9 ng/ml). The sensitivity, specificity, PPV and NPV were as follows: CA125 (83.3%; 75%; 80.6%; 78.3%), HE4 (86.6%; 91.3%; 92.9%; 84%) and YKL-40 (75%; 83.3%; 84%; 74.1%).

**Conclusion:**

Among the tested proteins the HE4 marker appears to be helpful in forecasting of optimal cytoreduction and possibly also of the prediction of response to platinum analogues used in first-line treatment of ovarian cancer.

## Introduction

Although the SEER report (National Cancer Institute Surveillance, Epidemiology, and End Results) shows a significant improvement in 5-year survival rate of patients with advanced ovarian cancer after the introduction of chemotherapy with paclitaxel and platinum analogues, the 10-year survival rate is still very low and is less than 10%. The generally accepted standard for ovarian cancer treatment at present consists in maximally cytoreductive surgery followed by cytotoxic adjuvant chemotherapy based on platinum and taxane compounds [1,2]. The main prognostic factor for patients with advanced ovarian cancer is maximal cytoreductive surgery, the maximum reduction in tumor size [3]. Unfortunately, not in all the patients with ovarian cancer it is possible to perform the primary, maximally cytoreductive oncological surgery without further exposing them to severe post-operative complications, while not meeting the requirement of no residual disease. Performing surgical procedures on primarily very advanced ovarian cancers may require the large intestine resection, resection of the diaphragm, spleen, partial liver resection. If at such a wide surgery the effect of complete cytoreduction is not achieved, the surgery being only a suboptimal procedure, the patient will not benefit from such a treatment, and the expected postoperative complications will delay the start of chemotherapy and consequently they will worsen the prognosis [4-6]. For about two decades the neoadjuvant chemotherapy has been successfully used in gynecologic oncology, in order to reduce the tumour mass and enable the optimal cytoreductive surgery after several courses of therapy [7]. The neoadjuvant chemotherapy is still of interest to physicians and scientists involved in the treatment of ovarian cancer. There are reports describing even a better prognosis for patients following the neoadjuvant chemotherapy [8], however the opinions are prevailing that it is a therapy by which similar long-term effects of treatment are achieved, as compared with the primary surgery [9]. It seems that the main problem is to isolate a group of women with ovarian cancer, which shall most benefit from treatment with neoadjuvant chemotherapy followed by interval debulking surgery. In the recent several years there have been scientific reports, aiming at identifying predictive factors for this group of patients [10,11]. These are research works both on imaging methods, investigating the biology of neoplastic tumours alone, as well as analysis of serous tumour markers [12-15].

The aim of the study was preliminary analysis if preoperative serum levels of CA 125, HE 4, YKL-40, bcl-2 and cathepsin-L could be helpful predictors for optimal cytoreductive surgery and response to platinum therapy in patients with malignant epithelial ovarian tumours.

## Materials and methods

To the study group the 70 consecutive patients with ovarian cancer were primarily enrolled. After analyzing the criteria for their inclusion, the final analysis was carried out on 56 patients diagnosed with ovarian cancer treated at the Department of Gynecological Surgery and Gynecological Oncology of Adults and Adolescents, Pomeranian Medical University.

The following criteria were adopted for inclusion:

○ diagnosed malignant epithelial ovarian cancer

○ having a full course of treatment with the first-line chemotherapy

○ a minimum follow-up six months after the last course of chemotherapy

○ expressing informed consent to the study

The exact characteristics of the patients are shown in Table 1.

**Table 1:** Patients demographics

**Mean age [range]**		
**56 [30–79]**		
	**n**	**%**
Premenopausal	12	21.4
Postmenopausal	44	78.6
FIGO I and II	17	30.4
FIGO III and IV	39	60.6
Grade 1	10	17.9
Grade 2	16	28.6
Grade 3	30	53.5
Serous type	45	80.4
Mucinous type	3	5.4
Endometriod type	3	5.4
Clear cell type	5	8.8
Primary optimal cytoreduction surgery	25	44.6
Neoadjuvant chemotherapy	31	55.4
Platinum sensitive	30	53.6
Platinum resistant	17	30.3
Platinum refractory	9	16.1

The study was of a retrospective character. Patients reporting at the clinic, who were suspected of ovarian cancer, signed their informed consents to the study and then had their blood samples taken on the day prior to the planned surgery. Serum was centrifuged and then it was frozen at temperature – 70°C until performing the tests HE4, YKL-40, cathepsin -L and bcl-2. The testing of each patient with CA 125 marker was performed in the preoperative period on date of blood sample taking.

Eligibility for surgery was based on clinical examination, CT or ultrasound imaging examinations. Patients who were suspected of very advanced neoplastic process and the likely inability to make a complete cytoreductive surgery were assessed as eligible for diagnostic laparoscopy, and in the absence of suspicion of high progression - to laparotomy. The necessity of conversion from laparoscopy to laparotomy due to the positive assessment of the complete resection ability of the neoplastic lesions occurred in 9 cases. On the other hand, as many as eight cases of laparotomy were of explorative character because the cytoreductive surgery failed to be performed. Finally, optimal debulking was achieved in 25 patients, and 31 were qualified as eligible for the neoadjuvant chemotherapy.

The patients after primary cytoreduction received six cycles of adjuvant chemotherapy with paclitaxel-carboplatin. Patients after primary investigative diagnostic surgery (laparoscopy or laparotomy) received 3–4 courses of neoadjuvant chemotherapy and then secondary cytoreductive surgery was performed on them, followed by subsequent courses of chemotherapy with paclitaxel-carboplatin.

The patients’ sensitivity to platinum analogues was evaluated according to the following criteria:

○ the platinum sensitive patients, if the disease-free time period was longer than 6 months after the end of the first-line chemotherapy

○ the platinum resistant patients, if the disease-free time period was shorter than 6 months after the end of the first-line chemotherapy

○ the platinum refractory patients, if disease progression occurred during the first-line chemotherapy

Progression of the disease was diagnosed on the basis of the value of CA 125 and CT results according to the RECIST criteria [16].

Due to the nature of the study, which focused on selected prognostic factors in women with ovarian cancer it was not possible to create a control group of volunteers with no history of ovarian cancer.

### Markers analysis

All tumour markers were performed in the Laboratory of Hormones and Tumour Markers at the Department of Gynecological Surgery and Gynecological Oncology of Adults and Adolescents, Pomeranian Medical University.

CA 125 levels were determined using CA125 immunoassay (EIA) system according to instructions from Abbott, (IMX, Abbott CA 125, Abbott Laboratories, Chicago, IL).

HE4 levels were determined using HE4 Fujirebio Diagnostics EIA assay. This solid-phase non-competitive immunoassay based on the direct sandwich technique was carried out according to the manufacturer’s instructions. The limit of detection was determined as < 2.5 pmol/l.

YKL40 levels were measured in serum using YKL40 ELISA kit from Metra Biosystems (Mountain View, CA).

Bcl-2 and Cathepsin L serum levels were measured using human ELISA kit from Bender Med Systems GmBH, Austria. These tests are for research use only. The limit detection of human Bcl-2 was determined to be < 0.5 ng/ml and for Cathepsin L 1.7 ng/ml.

### Statistic analysis

The average values of HE4, CA125, YKL-40, bcl-2 and cathepsin-L were compared between the group of patients who underwent primary surgery and the group of patients selected as eligible for neoadjuvant chemotherapy and between groups of women that were created according to the response to the first-line treatment. The statistical analysis was performed using STATISTICA 9.1 PL and PQStat 1.4.6. programs. The average values in each group were compared using nonparametric Mann–Whitney U test and Kruskal-Wallis test. Receiver operating characteristic (ROC) curves were obtained, and the area under curve (AUC) was calculated with 95% confidence interval according to the nonparametric method of DeLong [17]. We used also this method to compare AUCs. The sensitivity, specificity, positive predictive value (PPV) and negative predictive value (NPV) were determined using cut-off value calculated using Youden and deLong method. The level of significance was taken as p < 0.05.

## Results

**Figure 1:**
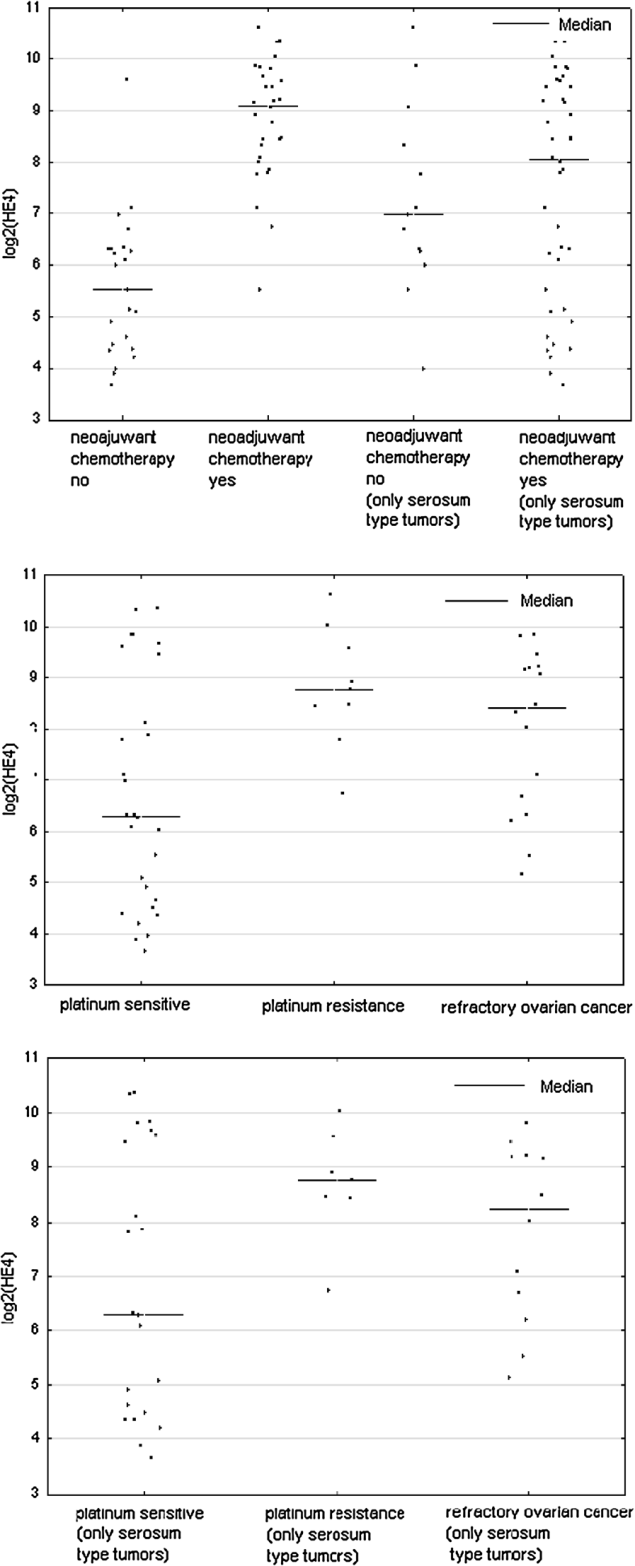
Scatterplot of serum HE4 levels for women with ovarian cancers according to primary treatment and platinum sensitivity.

**Figure 2:**
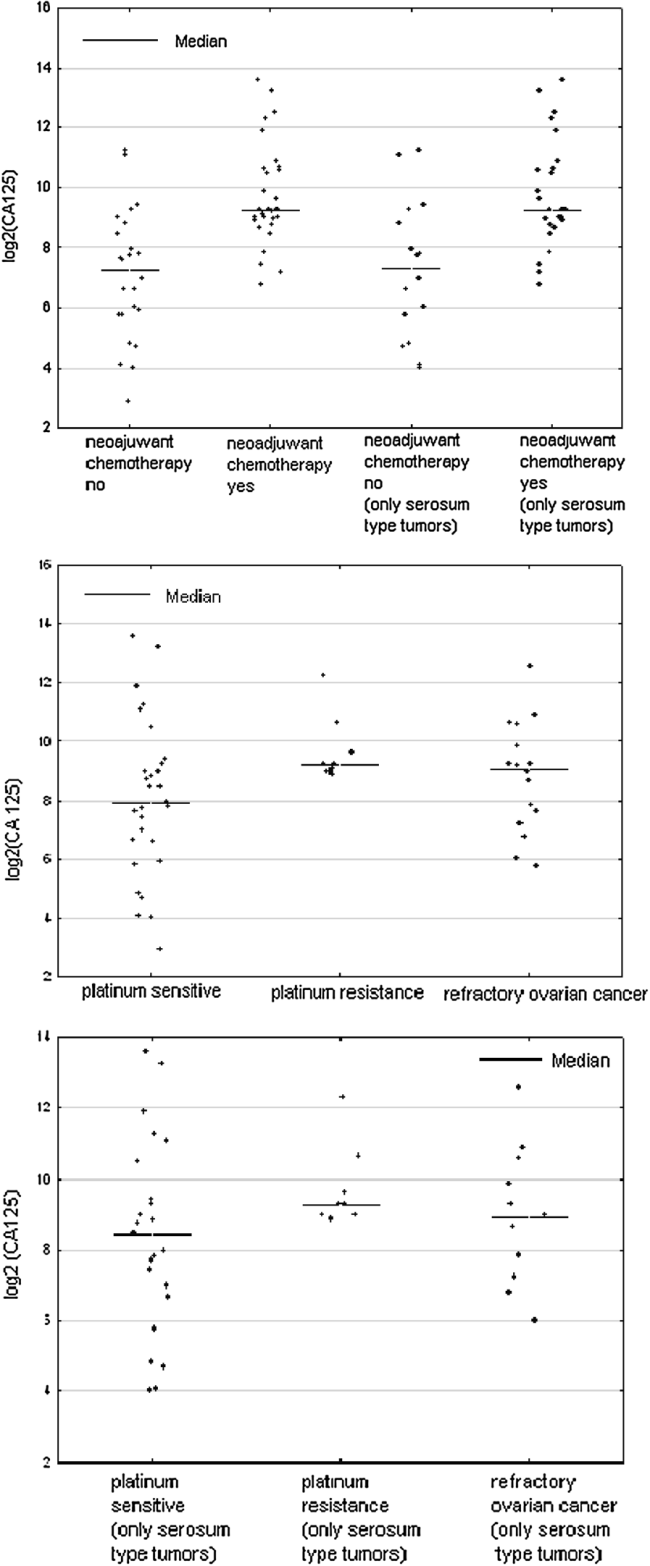
Scatterplot of serum CA 125 levels for women with ovarian cancers according to primary treatment and platinum sensitivity.

**Figure 3:**
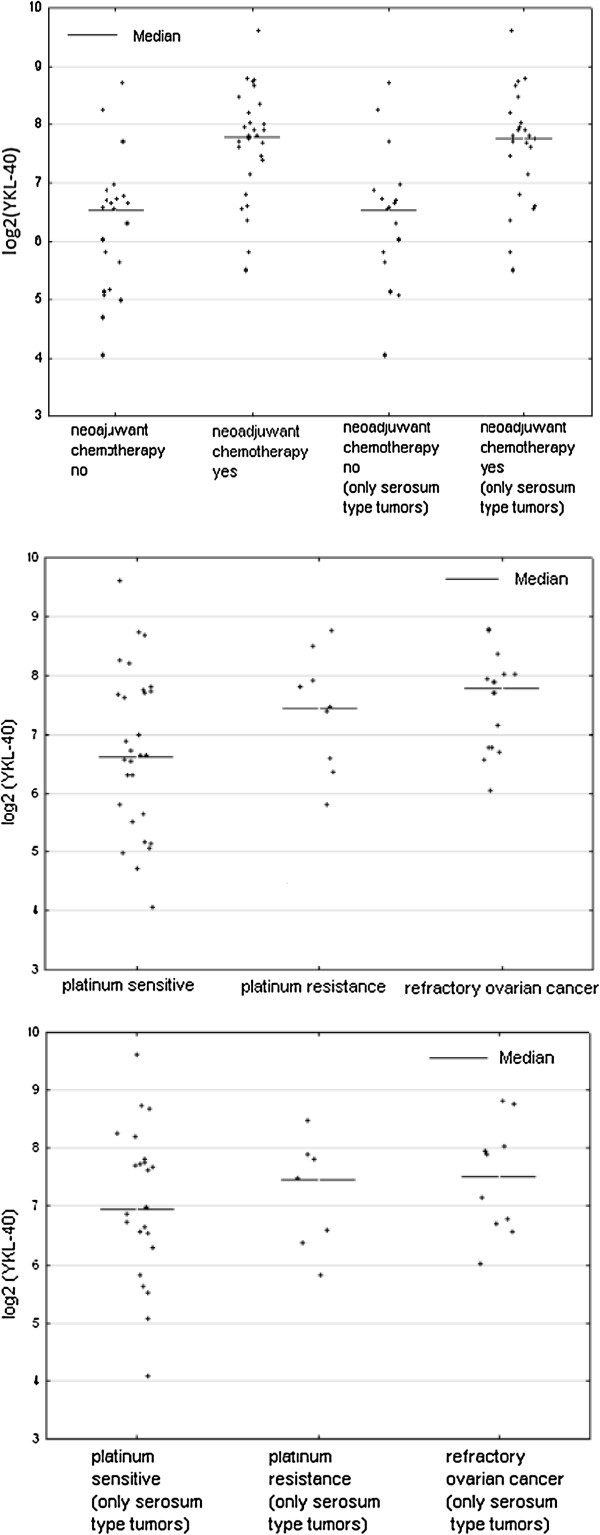
Scatterplot of serum YKL-40 levels for women with ovarian cancers according to primary treatment and platinum sensitivity.

When comparing the mean values of the concentrations of selected tumor markers (CA125, HE4, YKL-40), cathepsin-L and bcl-2 we found that statistically significant differences between a group of women undergoing primary surgical treatment and the patients qualified as eligible for the neoadjuvant chemotherapy were applicable only to the tumor markers (CA125 1206.79 vs 2432.38, p = 0.000191; HE4 78.87 vs 602.45, p = 0.000004; YKL-40 108.13 vs 203.96, p = 0.003991). Average values of cathepsin-L and Bcl-2 were similar in both groups and statistically insignificant (Table 2). By limiting the study group only to serous ovarian cancers we received very similar results (Table 3). Comparison of average concentrations of proteins studied by us, depending on the response to the first-line chemotherapy, are shown in Table 4. We found statistically significant differences in the mean concentrations of the tumour markers CA 125 and HE4 between the group of patients sensitive and resistant to the first-line chemotherapy, and these values were as follows, respectively (CA 125 575.77 vs 1338.79, p = 0.038, HE4 254.42 vs 566.22, p = 0.045). In the case of the average values of YKL-40 a statistically significant difference was observed between the group of women sensitive to platinum analogues and treatment refractory (131.04 vs 231.02, p = 0.0218). We also made a comparison between a group of platinum sensitive patients with platinum resistant and platinum refractory patients together (IS + R). In this comparison, the values of CA 125 and YKL-40 were significantly statistically higher in the group of refractory and resistant cases as compared with cases of ovarian cancer sensitive to chemotherapy based on platinum (CA 125 1058.15 vs 575.77, p = 0.0324, YKL-40 193.53 vs 131.04, p = 0.0339). There were no differences found in serum concentrations of cathepsin-L and Bcl-2 in the compared groups. By limiting the study group only to serous ovarian cancers it was found that only in the case of CA 125 the average values are higher in a group of women with resistant ovarian cancer as well as resistant and refractory jointly, as compared with the cancer sensitive to the first-line treatment, and these values were as follows, respectively: 1338.78 vs 656.25, p = .0258 and 1171 vs 656.25, p = 0.0451. In the case of HE4, YKL-40, cathepsin-L and bcl-2 the differences were not statistically significant. At Figures 1, 2, 3 there is a graphic presentation of the scatter of the values of the studied tumour markers, depending on the treatment applied and depending on the response to the first-line chemotherapy. The differences in the median values for HE4, CA 125 and YKL-40 are clearly visible, which are greater in women who start treatment with neoadjuvant chemotherapy as compared with patients that primarily underwent radical surgical procedures. Using the Youden method and the DeLong method the cut-off values were determined for the markers CA125, HE4 and YKL-40, which would identify the patients being the candidates for treatment with neoadjuvant chemotherapy. The following cut off point values were established: CA125-345 mIU/ml, HE4-218.43 pmol/ L and YKL-40-140.9 ng/ml. Based on these cut-off points and the contingency table, the sensitivity, specificity, positive and negative predictive values [PPV and NPV] were calculated for the tested markers, which were as follows: CA125 (83.3%; 75%; 80.6%; 78.3%), HE4 (86.6%; 91.3%; 92.9%; 84%) and YKL-40 (75%; 83.3%; 84%; 74.1%).

**Table 2:** Preoperative markers, bcl-2 and cathepsin–L serum concentrations depending on the method of treatment

	**Primary optimal cytoreduction**	**Neoadjuvant chemotherapy**	**p**
CA125 [IU/ml]			
Mean	1206.79	2432.28	
Median	190.7	600	p = 0.000191
Range	(7.38-21442)	(49.22-22982)	
HE4 [pmol/l]			
Mean	78.87	602.45	
Median	32.19	551.69	p = 0.000004
Range	(2.5-775.79)	(45.82-1574.89)	
YKL-40 [ng/ml]			
Mean	108.13	203.96	
Median	70.50	200.24	p = 0.003991
Range	(16.42-417.27)	(45.02-437.01)	
Cathepsin-L [ng/ml]			
Mean	13.12	12.75	
Median	12.64	11.05	p = 0.5305
Range	(8.42-28.87)	(8.53-46.45)	
bcl-2 [ng/ml]			
Mean	20.86	22.75	
Median	19.63	19.34	p = 0.5741
Range	(17.52-24.19)	(4.88-81.02)	

**Table 3:** Preoperative markers, bcl-2 and cathepsin–L serum concentrations depending on the method of treatment at serous tumors only

	**Primary optimal cytoreduction**	**Neoadjuvant chemotherapy**	**p**
CA125 [IU/ml]			
Mean	455.1	2661.46	
Median	167.25	600.0	p = 0.0019
Range	(15.81-2377.0)	(49.22-22982.0)	
HE4 [pmol/l]			
Mean	87.62	560.7	
Median	24.78	564.72	p = 0.0009
Range	(2.5-775.79)	(45.82-1310.69)	
YKL-40 [ng/ml]			
Mean	128.73	203.37	
Median	84.23	200.24	p = 0.005
Range	(16.42-417.27)	(45.02-437.01)	
Cathepsin-L [ng/ml]			
Mean	12.95	12.81	
Median	84.23	10.98	p = 0.8125
Range	(8.42-28.87)	(8.53-46.45)	
bcl-2 [ng/ml]			
Mean	12.01	23.98	
Median	20.98	20.07	p = 0.6913
Range	(11.27-45.53)	(4.88-81.02)	

**Table 4:** Preoperative markers, bcl-2 and cathepsin–L serum concentrations depending on response to chemotherapy

	**S**	**IS**	**p**	**S**	**R**	**p**	**IS**	**R**	**p**	**S**	**IS+R**	**p**
CA125 [IU/ml]			p = 0.038			p = 0.1852			p = 0.2261			p = 0.0324
Median	575.77	1338.79		575.77	949.01		1338.79	949.01		575.77	1058.15	
Range	(7.38-2743.5)	(464.43-4882.06)		(7.38-2743.5)	(49.23-22982)		(464.43-4882.06)	(49.23-22982)		(7.38-2743.5)	(49.23-22982)	
HE4 [pmol/l]			p = 0.045			p = 0.268			p = 0.127			p = 0.0888
Median	254.42	566.22		254.42	271.32		566.22	271.32		254.42	349.96	
Range	(2.5-1310.69)	(106.96-1574.89)		(2.5-1310.69)	(2.5-590.13)		(106.96-1574.89)	(2.5-590.13)		(2.5-1310.69)	(2.5-1574.89)	
YKL-40 [ng/ml]			p = 0.062			0 = 0.0218			p = 0.2452			p = 0.0339
Median	131.04	143.55		131.04	231.02		143.55	231.02		131.04	193.53	
Range	(16.42-417.27)	(55.06-235.01)		(16.42-417.27)	(63.98-437.01)		(55.06-235.01)	(63.98-437.01)		(16.42-417.27)	(55.06-437.01)	
Cathepsin-L [ng/ml]			p = 0.3242			p = 0.1734			p = 0.2759			p = 0.3461
Median	12.72	12.26		12.72	11.68		12.26	11.68		12.72	11.84	
Range	(8.42-46.45)	(9.74-14.98)		(8.42-46.45)	(9.34-18.17)		(9.74-14.98)	(9.34-18.17)		(8.42-46.45)	(9.34-18.17)	
bcl-2 [ng/ml]			p = 0.1010			p = 0.0719			p = 0.1073			p = 0.2777
Median	24.37	32.48		24.37	16.73		32.48	16.73		24.37	20.84	
Range	(18.91-29.84)	(9.89-81.02)		(18.91-29.84)	(4.88-30.94)		(9.89-81.02)	(4.88-30.94)		(18.91-29.84)	(4.88-81.02)	

S-platinum sensitive; IS-platinum resistant; R-platinum refractory.

**Figure 4:**
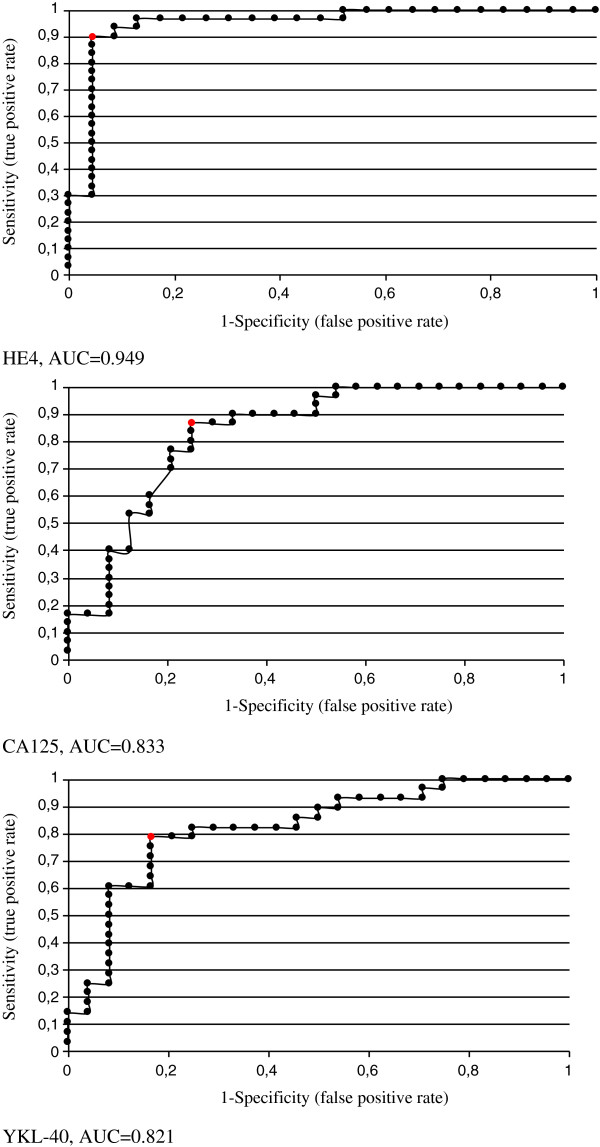
ROC curves of HE4, CA 125 and YKL-40 at all tumours.

**Figure 5:**
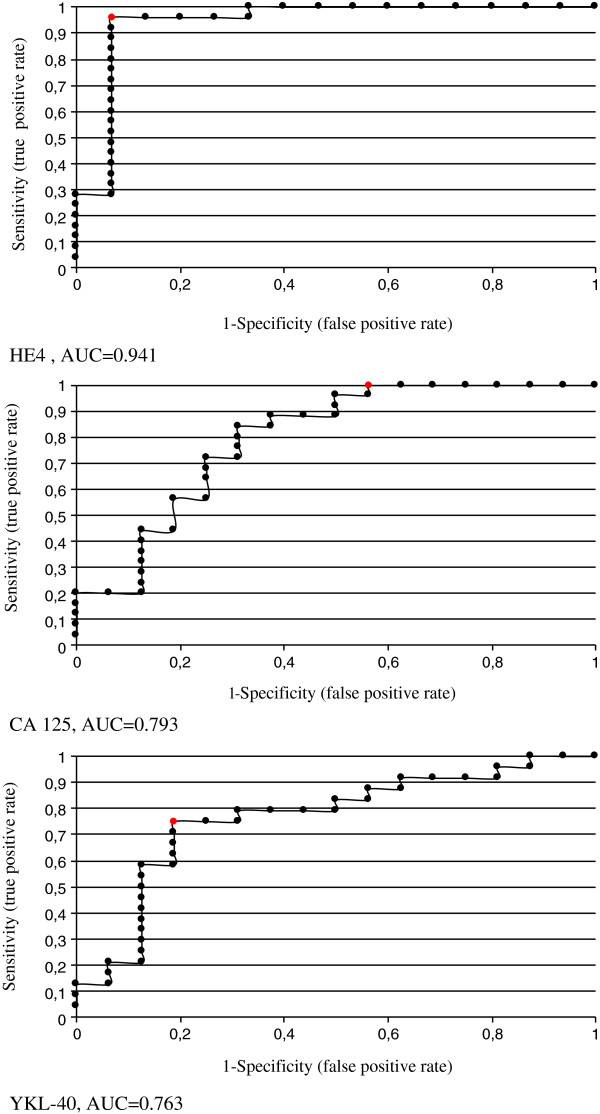
ROC curves of HE4, CA 125 and YKL-40 at serous type tumours.

The Figures 4 and 5 show the ROC curves for CA 125, HE4 and YKL-40 as the markers preliminarily discriminating patients with ovarian cancer into the cases that are candidates for primary cytoreduction or not. The AUC values, both within the entire group and in the group of only serous carcinomas are the highest for HE4 (0949 and 0941). When comparing values of the area under the curve (AUC) for the tested markers, in most cases no statistically significant differences were found, besides the superiority of HE4 over YKL-40 in the serous cancer group. For the whole study group: AUC for HE4 (0.949) vs CA 125 (0.833), p = 0.073, HE4 (0.949) vs YKL-40 (0.821), p = 0.088, CA125 (0.833) vs YKL-40 (0.821), p = 0.94. For the serous cancer group: AUC for HE4 (0.949) vs CA 793 (0.833), p = 0.073, HE4 (0.949) vs YKL-40 (0.821), p = 0.088, CA125 (0.833) vs YKL-40 (0.821), p = 0.94.

No cut-off points were determined in groups of patients analyzed in terms of sensitivity to platinum analogues. The constructed ROC curves did not meet the criteria of a valuable diagnostic test, as the areas under the curve (AUC) for the CA 125, HE4 and YKL-40 were in each case less than 0.5.

## Discussion

In view of the rather numerous recent studies, it appears that the neoadjuvant chemotherapy is a valuable option for patients who cannot have the surgery with optimal cytoreduction performed on them. A certain problem could be the classification of patients into those being eligible for an appropriate treatment option. In our work, we made an initial attempt to assess the potential usefulness of preoperative serum levels of commonly known markers for predicting the optimal therapeutic regimen: cytoreductive surgery followed by chemotherapy or chemotherapy followed by cytoreductive surgery. In addition, we assessed the relation of these markers with response to the first-line chemotherapy.

CA125 is the most studied tumor marker for ovarian cancer, known for its diagnostic and prognostic usefulness in malignant epithelial ovarian cancers. Many works also report on the usefulness of this marker in the use of the neoadjuvant chemotherapy on many levels [14,15,18] but there are also authors who question its relevance in the said therapeutic process [19,20]. Of interest are studies carried out by Kang et al. [14], who studied 314 women with ovarian cancer and found that the CA 125 can be a very good predictor of selecting a suitable therapeutic option at the start of the treatment of ovarian cancer. The authors compared a group of patients undergoing the primary cytoreductive surgery (n = 220) with patients treated with neoadjuvant chemotherapy followed by secondary cytoreduction (n = 94). The initial results of the comparison of both groups showed no difference in time to disease progression (PFS), but by analyzing a group treated with the neoadjuvant chemotherapy and analyzing its various subgroups (age above/below 65 years, stage, histopathological type and level of CA 125) it turned out that it is just the marker level that is a key factor. Patients having an baseline value of CA 125 above 2000 U/ml and qualified as eligible for the neoadjuvant chemotherapy gain significant advantages in terms of longer time to progression (HR 0.62, 95% CI 04 to 0.97, p = 0.037). Gąsowska-Bodnar et al. [18] showed, however, that the regression coefficient of CA 125 after two courses of the neoadjuvant chemotherapy has statistically significant effect on progression-free time (TTS- time to survival). Rocconi et al. [21] presented a study in which early normalization of the CA 125 marker during chemotherapy was associated with a greater sensitivity to platinum analogues with a longer time to progression (PFS) and overall survival time. The CA 125 as a strong predictor of optimal cytoreduction was also described in studies conducted by Vasudev et al. [22]. AUC in their research for CA 125 as a predictor of optimal cytoreduction was 0756. In our study we manifested the usefulness of CA 125 in predicting the need of the neoadjuvant chemotherapy in a selected group of patients with advanced ovarian cancer. The area under the curve (AUC) for CA 125 was 0.833 for the entire study group and 0.793 for the serous carcinomas group which is consistent with studies carried out by Vasudev et al. [22]. We also showed a statistically significant difference in baseline values of CA 125 among a group of women undergoing the primary surgery followed by the neoadjuvant chemotherapy and between the sensitive and resistant patients to platinum analogues. Our study confirmed the usefulness of CA 125, previously described in the literature, in the neoadjuvant chemotherapy prediction.

HE4 is a new tumour marker, recently approved for the diagnosis and monitoring of ovarian cancer. Human epididymis protein 4 (HE4) is a member of the “four-disulfide core” family, heterogeneous group of small acid- and heat-stable proteins of biological function not yet detailed identified. A hypothesis was advanced that the WFDC2 protein may be a component of the innate immune response in the lung, nose and oral cavity [23,24]. By immunohistochemical examination the expression of HE4 was found in a normal epithelium of the genital tract in women, serous, endometrioid and clear cell type neoplastic ovarian tumors [25]. Data on the role of HE4 in the carcinogenesis are inconsistent. Gao et al. [26] demonstrated that the transfer of “exogenous HE4 gene” to the ovarian cancer cell lines significantly promotes cell apoptosis and it can contribute to the protective role of this gene in the ovarian cancer progression process. Zou et al. [27] found out that the HE4 gene silencing results in cell division stopping in the G0/G1 phase which in turn is associated with the inhibition of proliferation, migration and invasion of the ovarian cancer cells. The HE4 participation in promoting the neoplastic tumor growth was also demonstrated by other authors, they demonstrated that the HE4 expression in cancer cells is associated with greater adhesion, migration and proliferation which may be dependent on the EGFR-MAPK cascade [28]. Knowledge of its usefulness in the various diagnostic and prognostic aspects of the ovarian cancer is still negligible. Only in recent years there have been published a lot of works associated with this marker [29-32]. Evaluation of usefulness of HE4 in predicting the success of cytoreductive surgery was analyzed in four publications, and all these publications confirmed its usefulness [15,31-34] Kalapotharakos et al. [31] found a significantly higher concentration of HE4 in a group of patients in which an attempt to perform cytoreductive surgery (578 pmol/L) failed as compared with patients who underwent radical surgery (278 pmol/L). In our study, the mean value of HE4 in the group of patients radically operated at the moment of diagnosis was 78.87 pmol/L and in the group treated with neoadjuvant chemotherapy 602.45 pmol/L. Yang et al. [33] showed that the 600 pmol/L is the cut-off value for HE4, above which a deferred cytoreductive surgery should be performed and the sensitivity and specificity of the test were 77 and 32%, respectively. In our study, the cut-off value for HE4 was lower and calculated based on the method of Youden was 218.43 pmol/L achieving a sensitivity and specificity at 86.6 and 91.3% levels, respectively. The cut-off value for HE4 (262 pmol/L) approximate to ours was presented by Angioli et. al. [15]. The sensitivity and specificity of the test varied then between 100 and 89.5% levels. They showed at the same time that HE4 in comparison with CA 125 is a better predictor of the feasibility of optimal cytoreduction, which was also confirmed in our study. In studies conducted by Braicu et al. [34] the 90% sensitivity was achieved in predicting incomplete cytoreduction if both markers CA 125 and HE4 had value exceeding 75 U/ml and 75 pmol/L. Assuming for HE4 the cut-off value at 235 pmol/L and 500 pmol/L levels they reported sensitivity and specificity at 76.6 and 47.3 levels as well as 51.9% and 70.4%, respectively. When they calculated the risk for incomplete cytoreduction using both markers with cut-off values of 235 pmol/L for HE4 and 500 U/ml for CA 125, the sensitivity was 64.8% and specificity 73.5%. The AUC for HE4 as a predictor of suboptimal surgery in ovarian cancer was 0.634 (34). In our study, the area under the curve was assessed for HE4 at 0.949 level for all the patients and 0.941 in the serous carcinomas group and these are the results that show a high potential of the marker HE4 in predicting the optimal cytoreduction which is consistent with all previous studies by other authors [15,31-33]. So far, no studies have been shown concerning the connection of HE4 with the platinum sensitivity in patients with ovarian cancer. In our study, however we found a significant difference in the values of HE4 in patient group sensitive (254.42 pmol/L), and resistant to chemotherapy (566.22 pmol/L), p = 0.045.

YKL-40 is a serum protein, whose elevated values occur in the course of inflammatory and neoplastic diseases. It was shown that the elevated values of YKL-40 in patients in the general population may be associated with an increased risk of developing colorectal cancer [35]. The immunoexpression in tissues is associated with increased proliferation. High expression of YKL-40 in conjunction with Ki-67 was showed in tissues of serous low-differentiated ovarian cancer [36]. According to Yip et al. [37] this marker is ranked on the 6^th^ position of the 175 tested compounds as potential markers for ovarian cancer. AUC for YKL-40 was 0.804, for CA 125 0.907 and for HE4 0.933. In our previous studies [38] we showed that YKL-40 is elevated in the serum of patients with ovarian cancer, however it did not demonstrate superiority over the CA 125. In the current study, we compared the usefulness of YKL-40 in predicting optimal cytoreduction. We found differences in serum concentrations in the compared groups, we carried out analysis for the diagnostic test determining the ROC curves and calculating the AUC, which was 0.821 for the whole group and 0.763 for serous carcinomas. Despite the high values of AUC, we did not demonstrate the superiority of YKL-40 over the CA 125 and especially over the HE4. Serum levels of YKL-40 in our study were significantly statistically higher in the group of women refractory to platinum analogues as compared with women sensitive to these drugs, which has not yet been described in the relevant literature.

The apoptosis process plays an important role in the carcinogenesis and is under the control of proteins from bcl-2 family [39]. Numerous studies suggesting an association of bcl-2 with the course of ovarian cancer were presented [39]. The relationship of the bcl-2 with the ovarian cancer progression stage was shown [40], with its usefulness in the early ovarian cancer detection [41] as well as the prognosis of the disease course [42,43]. Research conducted by Crasta et al. [44] showed that the factor associated with suboptimal cytoreduction is the degree of angiogenesis intensification and at the same time a negative correlation between bcl-2 and VEGF was demonstrated. Dutta et al. [45] reported that chemotherapy leads to increased apoptosis by independent p-53 way, causing a reduction of bcl-2 protein and survivin but not bcl-XL. It was further found that applying 6 courses is more effective in the neoadjuvant chemotherapy than 3 courses in terms of induction of apoptosis [45]. In other reports it was shown that high expression of bcl-2 has an adverse effect on the occurrence of complete remission, but only in the cancer group TP 53 negative [46]. In our studies, in contrast to studies conducted by Camlica et al. [47], we did not show any differences in preoperative serum values of bcl-2 in both tested groups, both in terms of optimal cytoreduction and sensitivity to platinum analogues. It does not appear that serum levels of bcl-2 may have the prognostic significance in this regard.

Cathepsin-L is a lysosomal endopeptidase belonging to the cysteine protease papain superfamily. It is synthesized as a proenzyme, then modified and after transport to lysosomes it performs many functions in the organism [48,49]. Physiologically it is involved in proteolysis and release of thyroxine and triodotironin, in the thyroid gland, it is involved in the immunological response by activating a number of processes that result in the formation of the MHC II molecules and consequently in antigen presentation. It also plays a role in the process of spermatogenesis as well as oogenesis and embryogenesis [48]. The share of cathepsin-L in pathological processes such as the neoplastic process is also postulated. It is probably involved in apoptosis and in cancers it is capable of hydrolyzing components of the extracellular matrix allowing tumour cells both local invasion and distant metastasis [48]. Cathepsin elevated levels were observed in serum of patients with ovarian cancer [49-52]. Nishida et al. [49] demonstrated that elevated cathepsin-L levels, especially while determining CA 125 and CA 72–4, can be a helpful method of detection of early ovarian cancer. Research conducted by Zhang et al. [50] showed that cathepsin-L, jointly with heparanase and matrix metalloproteinase-9 correlate with malignant invasion and progression in ovarian cancer and their combined determination can be a good predictor of the presence of metastases prior to the planned surgery. Similar results were presented by Kolwijck et al. [53], as well as by Wang et al. [52]. The values of cathepsin L obtained in the research conducted by Zhang et al. [50] are as follows: the mean value in patients with ovarian cancer 21.23 ng/ml, in healthy patients 5.59 ng/ml, in advanced ovarian cancer 22.6 ng/ml and at the FIGO stage I-II 19.6 ng/ml. The AUC value was 0.708 for cathepsin L and 0.776 for CA 125. In our study there was no difference in the values of cathepsin-L between the study groups. However, in each tested group the cathepsin-L values were higher than expected in healthy patients group (according to manufacturer the average expected values are about 5 ng/ml). The same value of cathepsin-L were observed preoperatively in patients initially treated with surgery vs. initially treated with chemotherapy as well as the same values regardless of the response to chemotherapy suggest that cathepsin-L cannot be probably a good predictor of patient eligibility for appropriate treatment groups.

After detailed analysis of the tested material, and in summing the results it appears that the most promising marker can be the HE4 marker both in forecasting the optimal cytoreduction in the primary surgical treatment of ovarian cancer as well as for predicting response to chemotherapy based on platinum analogues. Further prospective studies on an exhaustive material are absolutely needed to confirm the results of the authors and of the cited research.

## Competing interests

The authors declare that they have no competing interests.

## Authors’ contributions

ACG have made substantial contributions to conception and design, planned and ran the experiments, collected data, performed analysis and interpretation of the results, review the literature and wrote the manuscript. ACP have contributed in collected data. JM have contributed in literature review. ASR supervised statistical analysis. ATG worked as pathologist. IRG have given final approval of the version to be published. All authors read and approved the final manuscript.
